# High-throughput neuroimaging-genetics computational infrastructure

**DOI:** 10.3389/fninf.2014.00041

**Published:** 2014-04-23

**Authors:** Ivo D. Dinov, Petros Petrosyan, Zhizhong Liu, Paul Eggert, Sam Hobel, Paul Vespa, Seok Woo Moon, John D. Van Horn, Joseph Franco, Arthur W. Toga

**Affiliations:** ^1^Laboratory of Neuro Imaging, Institute for Neuroimaging and Informatics, University of Southern CaliforniaLos Angeles, CA, USA; ^2^Biomedical Informatics Research Network, Information Sciences Institute, University of Southern CaliforniaLos Angeles, CA, USA; ^3^Statistics Online Computational Resource, University of Michigan, UMSNAnn Arbor, MI, USA; ^4^Department of Computer Science, University of CaliforniaLos Angeles, Los Angeles, CA, USA; ^5^Brain Injury Research Center, Department of Neurosurgery, David Geffen School of Medicine, University of CaliforniaLos Angeles, Los Angeles, CA, USA; ^6^Department of Neuropsychiatry, Konkuk University School of MedicineSeoul, Korea

**Keywords:** aging, pipeline, neuroimaging, genetics, computation solutions, Alzheimer's disease, big data, visualization

## Abstract

Many contemporary neuroscientific investigations face significant challenges in terms of data management, computational processing, data mining, and results interpretation. These four pillars define the core infrastructure necessary to plan, organize, orchestrate, validate, and disseminate novel scientific methods, computational resources, and translational healthcare findings. Data management includes protocols for data acquisition, archival, query, transfer, retrieval, and aggregation. Computational processing involves the necessary software, hardware, and networking infrastructure required to handle large amounts of heterogeneous neuroimaging, genetics, clinical, and phenotypic data and meta-data. Data mining refers to the process of automatically extracting data features, characteristics and associations, which are not readily visible by human exploration of the raw dataset. Result interpretation includes scientific visualization, community validation of findings and reproducible findings. In this manuscript we describe the novel high-throughput neuroimaging-genetics computational infrastructure available at the Institute for Neuroimaging and Informatics (INI) and the Laboratory of Neuro Imaging (LONI) at University of Southern California (USC). INI and LONI include ultra-high-field and standard-field MRI brain scanners along with an imaging-genetics database for storing the complete provenance of the raw and derived data and meta-data. In addition, the institute provides a large number of software tools for image and shape analysis, mathematical modeling, genomic sequence processing, and scientific visualization. A unique feature of this architecture is the Pipeline environment, which integrates the data management, processing, transfer, and visualization. Through its client-server architecture, the Pipeline environment provides a graphical user interface for designing, executing, monitoring validating, and disseminating of complex protocols that utilize diverse suites of software tools and web-services. These pipeline workflows are represented as portable XML objects which transfer the execution instructions and user specifications from the client user machine to remote pipeline servers for distributed computing. Using Alzheimer's and Parkinson's data, we provide several examples of translational applications using this infrastructure^1^.

## Introduction

The long-term objectives of computational neuroscience research are to develop models, validate algorithms and engineer powerful tools facilitating the understanding of imaging, molecular, cellar, genetic, and environmental associations with brain circuitry and observed phenotypes. Most of the time, functioning teams of interdisciplinary investigators are necessary to develop innovative approaches to substantively expand the ways by which brain structure and function can be imaged in humans. Prototype development, proof of concept pilot studies and high-risk, high-impact research requires substantial infrastructure to support the data management, processing and collaboration.

There are significant barriers that inhibit our ability to understand the fundamental relations between brain states and the wide spectrum of observable, direct and indirect, biological, genetic, imaging, clinical, and phenotypic markers. Some of these challenges pertain to lack of models and algorithms for representing heterogeneous data, e.g., classifying normal and pathological variation (biological noise vs. technological errors) (Liu et al., 2012; Sloutsky et al., 2013). Others are driven by limitations in the available hardware and infrastructure resources, e.g., data size and complexity, data management, and sharing logistics, Distributed processing and data mining (Dinov et al., 2013; Kandel et al., 2013; Van Horn and Toga, 2013).

There are a number of teams and ongoing efforts that develop computational infrastructures to address specific research needs. For instance, the efforts of the Enhancing Neuroimaging Genetics through Meta-Analysis (ENIGMA) consortium (http://enigma.loni.usc.edu) represents a collection of World-wide research groups which agreed on a social networking strategy for data aggregation and sharing (Novak et al., 2012). ENIGMA manages imaging and genomics data facilitating the process of understanding brain structure and function using structural, functional, diffusion imaging and genome-wide association study (GWAS) data. The network's goal is to enable meta-research and replicated findings via increasing sample-sizes (cf. statistical power to detect phonotypic, imaging or genetic effects) in a collaborative fashion where investigators and groups share algorithms, data, information, and tools.

The high-throughput analysis of large amounts of data has become the ubiquitous norm in many computational fields, including neuroimaging (Barker and Van Hemert, 2008; Barrett et al., 2009; Dinov et al., 2009). The driving forces in this natural evolution of computerization and protocol automation are parallelization, increased network bandwidth, and the wide distribution of efficient and potent computational and communication resources. In addition, there are now more and larger data archives, often accumulating many hundreds, if not thousands, of subjects with enormous amounts of data. These can only be processed using efficient and structured systems. Efficient and effective tool interoperability is critical in many scientific endeavors as it enables new types of analyses, facilitates new applications, and promotes interdisciplinary collaborations (Dinov et al., 2008). The Pipeline Environment (Rex et al., 2003; Dinov et al., 2009) is a visual programming language and execution environment that enables the construction of complete study designs and management of data provenance in the form of complex graphical workflows. It facilitates the construction, validation, execution, and dissemination of analysis protocols, computational tools, and data services. The Pipeline has been used to construct advanced neuroimaging protocols analyzing multi-subject data derived from the largest publically available archives, including the International Consortium for Brain Mapping (ICBM) (Mazziotta et al., 1995), Alzheimer's Disease Neuroimaging Initiative (ADNI) (Mueller et al., 2005), Australian twin data of brain activation and heritability (Blokland et al., 2008), British infant database (Gousias et al., 2008), and the MNI (Evans, 2006) pediatric database.

Other significant efforts to provide computational infrastructure for high throughput brain data analyses include Taverna (Oinn et al., 2005) http://www.taverna.org.uk, Kepler (Ludäscher et al., 2006) kepeler-project.org, Khoros (Kubica et al., 1998), www.khoral.com, Trident Workbench (Toga et al., 2012), http://tridentworkflow.codeplex.com, Karma2 (Simmhan et al., 2008), http://www.extreme.indiana.edu/dist/java-repository/workflow-tracking/, Galaxy (Goecks et al., 2010), http://galaxy.psu.edu, and many others.

Examples of significant scientific, computational, and analytic challenges include:

1. *Software Tool Interoperability:* Differences in software development strategies can force intrinsic incompatibilities in algorithm design, implementation strategy, data format, or tool invocation syntax. For example, there are data type, array management, and processing differences in different language platforms, which complicate the integration of inputs and outputs. There also can be variations in implicit and explicit parameter specifications and services vs. command-line invocation syntax. The Distributed Pipeline addresses this barrier by providing an extensible markup language protocol for dynamic interoperability of diverse genomics data, informatics software tools, and web-services.
2. *Hardware Platform Dependencies:* Processor endianness (e.g., byte-swaps), architectural differences (e.g., 32 vs. 64-bit), compiler variations, and security incompatibilities cause significant problems in the integration of data and computational resources residing on multiple platforms. These hardware idiosyncrasies limit the potential to utilize the most appropriate computational resources on multi-platform systems and reduce the efficiency of many computational approaches. The distributed Pipeline server will provide a native and virtualized environment for configuring, deploying, and running Distributed Pipeline on different hardware platforms.
3. *Data Heterogeneity:* Biological data often include heterogeneous information, such as clinical, genetic, phenotypic, and imaging data. Moreover, these data can be large (often measured in Gigabytes). These two characteristics necessitate care in the design and execution of data processing protocols. Frequently, the processing of heterogeneous data is performed by independent analyses within each data type followed by *ad hoc* strategies for integration, visualization, and interpretation. For instance, neuroimaging genetics studies (Ho et al., 2010a,b) utilize imaging, genetic, and phenotypic data, but most bioinformatics data analysis tools enable processing of only uni-modal spatiotemporal, sequence, or spreadsheet type data. The joint modeling and analysis of such multiform data will significantly increase our ability to discover complex associations, biomarkers, and traits that are currently implicit in the complex genomics data. The Distributed Pipeline study-design mechanism will enable the integration of imaging and meta-data as well as the construction of complete study protocols using the entire data collection. For instance, Distributed Pipeline will enable dynamic decision making, branching, and looping based on the meta-data and on data derived in the analysis protocol itself. Another data-related challenge includes anonymization and/or de-identification of hosted data, to comply with IRB/HIPAA regulations, protect personal information and ensure subject privacy. The LONI/INI infrastructure provides a two-tier mechanism for data de-identification. First the Imaging Data Archive system ensures that all data (imaging, genetic, demographic) submitted to the database excludes all personal identifiable information (http://www.loni.usc.edu/Software/DiD). Second, the Pipeline environment provides customizable modules for data anonymization, which can be included in the beginning of any graphical processing workflow to ensure the protocol generates intermediate and final results excluding personal information.
4. *Result Reproducibility:* Genomics and informatics protocol dissemination, study replication, and reproducibility of findings have become increasingly important in scientific investigation. Dissemination includes technical publications, distribution of data, URL links, software tools, and execution scripts, as well as screencast, videos, tutorials, and training. Most of these methods for distribution of novel research protocols do not enable outside investigators to independently and efficiently test, validate, or replicate newly proposed techniques. As a result, investigators may frequently reinvent analysis protocols, fail to follow exact procedures, or misinterpret alternative findings. Even when there is a clear description of the scientific model employed in a study (e.g., general linear model), there may be differences in the algorithmic implementation, hardware platform, compiler, environment configuration, or execution-syntax, which can cause differences in the results even using the same input data. The Distributed Pipeline infrastructure will enable flexible and efficient distribution of published (peer-reviewed) workflows, which will facilitate result reproducibility and validation of analysis protocols by the entire user community. Previously developed, validated and published workflows are available online (http://pipeline.loni.usc.edu/explore/library-navigator/).
5. *Steep Learning Curve:* Other informatics challenges include steep learning curves for utilizing general distributed computing environments, and incompatible differences in communication protocols. Currently, significant technical knowledge is required to configure, utilize, and link diverse sequence analysis tools. This task is typically done by developing sophisticated scripts and/or repackaging software resources within specific graphical workflow environments. The Distributed Pipeline computational library will contain a large number of data references and software resources. The included XML data, module, and workflow descriptions will abstract many of the technical details about the standard and advanced features of these resources and promote appropriate access, easy use and efficient modification of the entire compendium of resources available within the Distributed Pipeline library.

Three notable successes include the Biomedical Informatics Research Network (BIRN), the International Neuroinformatics Coordinating Facility (INCF) and the cancer Biomedical Informatics Grid (caBIG). BIRN is a national initiative focused on advancing biomedical research through data sharing and online collaboration. It is funded by the National Institute of General Medicine Sciences (NIGMS), and provides data-sharing infrastructure, software tools, strategies and advisory services—all from a single source (Keator et al., 2008). INCF supports a collaborative neuroinformatics infrastructure and promotes the sharing of data and computing resources to the international research community. INCF is funded by contributions from its member countries, based on gross domestic expenditures on research and development (GERD), www.incf.org. The caBIG program developed and supports access to digital capabilities essential to enhancing researchers' capacity to utilize biomedical information. The initiative aims to disseminate and promote the use of open source standards for data exchange and interoperability in cancer research, develop, maintain, enhance, and share innovative biomedical informatics capabilities, and facilitate the management and analysis of big and heterogeneous cancer research data sets (von Eschenbach and Buetow, 2006).

In this paper, we present the novel infrastructure at the USC Institute for Neuroimaging and Informatics, which is available to the entire computational neuroscience community and addresses many of the current computational neuroscience barriers—lack of integrated storage, hardware, software and processing Big Data infrastructure, limitations of current infrastructure for processing of complex and incomplete data, and the difficulties with resource interoperability.

## Resource infrastructure

The INI provides an extensive infrastructure designed and operated to facilitate modern informatics research and support for hundreds of projects including several multi-site national and global efforts. We have redundancies built in to all equipment, and a secure facility to protect equipment and data. The resources described below provide networking, storage and computational capabilities that will ensure a stable, secure and robust environment. It is an unprecedented test bed to create and validate big data solutions. Because these resources have been designed, built and continuously upgraded over the years by our systems administration team, we have the appropriate expertise and operating procedures in place to use these resources to their maximum benefit.

The INI/LONI data center contains a 300 KVa UPS/PDU capable of providing uninterruptible power to mission-critical equipment housed in the room, dual 150 KVa connections to building power, an 800 kW Caterpillar C27 diesel backup generator, three Data Aire computer room air conditioning (CRAC) units, humidity control, and a Cisco fire suppression and preaction system. A sophisticated event notification system is integrated in this space to automatically notify appropriate personnel of any detrimental power and HVAC issues that arise.

### Data center security

The LONI datacenter is secured by two levels of physical access, to insure HIPAA compliance for data security. The main facility is secured 24/7 with access control devices. Only authorized personnel are allowed in, and guests are permitted only after checking in, and only during business hours. The datacenter itself is additionally secured by a second layer of proximity card access. Only authorized staffs are permitted to enter the datacenter facility. Individual racks containing HIPAA data are secured by lock and key to prevent cross access.

### Computational and storage resources

**Figure 1:**
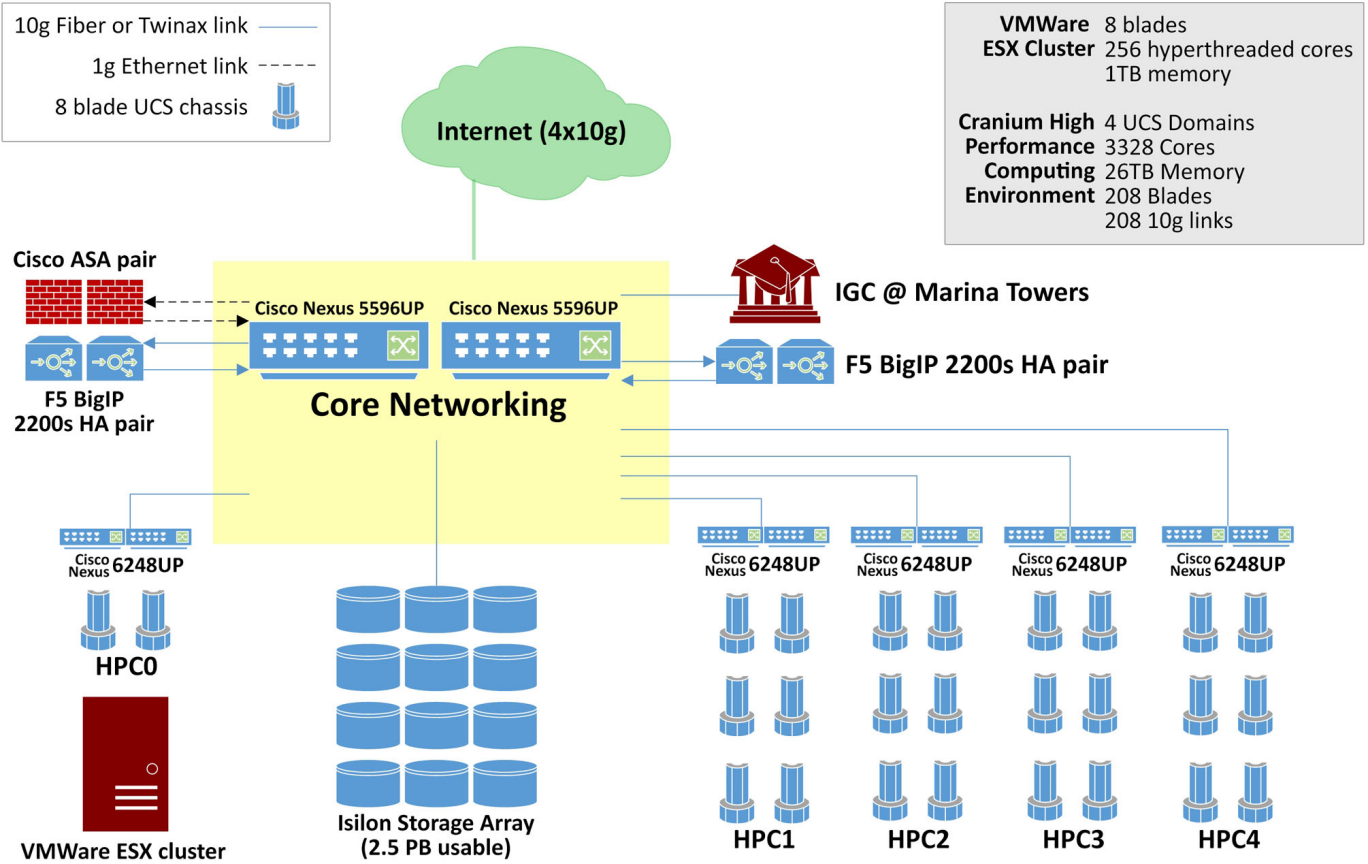
**LONI/INI network infrastructure and supercomputing environnement**.

Rapid advancements in imaging and genetics technology have provided researchers with the ability to produce very high-resolution, time-varying, multidimensional data sets of the brain. The complexity of the new data, however, requires immense computing capabilities. The compute infrastructure within the datacenter boasts 3328 cores and 26 Tb of aggregate memory space, Figure 1. This highly available, redundant system is designed for demanding big data applications. Blades in the Cisco UCS environment are easy to replace. A failing blade sends an alert to Cisco where a replacement ticket is generated automatically. Upon arrival, the new blade can go from the shipping box to being fully provisioned and in production in as little as 5 min. Institutions and scientists worldwide rely on the LONI's resources to conduct research. LONI is architected using a fault-tolerant, high-availability systems design to ensure 24/7 functionality. The primary storage cluster is 23 Isilon nodes with 2.4 usable petabytes of highly available, high performance storage. Data in these clusters moves exclusively over 10 g links excepting node to node communication in the Isilon cluster which is handled by QDR Infiniband, providing 40 gigabit bidirectional throughput on each of the Isilon cluster's 46 links. Fault tolerance is as important as speed in the design of this datacenter. The Isilon storage cluster can gracefully lose multiple nodes simultaneously without noticeably affecting throughput or introducing errors.

External services are load balanced across four F5 BIG-IP 2200S load balancers. The F5 load balancers provide balancing services for web sites, applications, as well as ICSA-certified firewall services. The INI core network is entirely Cisco Nexus hardware. Each of the two Cisco Nexus 5596 s supports 1.92 Tb per second of throughput. Immediately adjacent to this machine room is a user space with twelve individual stations separated by office partitions. These workspaces are manned by staff who constantly monitor the health of the data center as well as plan for future improvements. Each space is also equipped with a networked workstation for image processing, visualization and statistical analysis.

### Network resources

Service continuity, deterministic performance and security were fundamental objectives that governed the design of LONI's network infrastructure. The laboratory intranet is architected using separate edge, core and distribution layers, with redundant switches in the edge and core for high availability, and with Open Shortest Path First (OSPF) layer 3 routing, instead of a traditional flat layer 2 design, to leverage the fault tolerance offered by packet routing and to minimize network chatter. While ground network connectivity is entirely Gigabit, server data connectivity is nearly all 10 Gb fiber and Twinax connected to a core of 2 Cisco Nexus 5596 switches, 10 Cisco Nexus 6628 switches, and 6 Cisco Nexus 2248 fabric extenders. For Internet access, INI is connected to the vBNS of Internet2 via quad fiber optic Gigabit lines using different route paths to ensure that the facility's external connectivity will be maintained in the case of a single path failure.

The facility has two Cisco Adaptive Security Appliances providing network security and deep packet inspections. LONI has also implemented virtual private network (VPN) services using SSLVPN and IPsec services to facilitate access to internal resources by authorized users. A VPN connection establishes an encrypted tunnel over the Internet between client and server, ensuring that communications over the Web are secure. Furthermore, the laboratory has an extensive library of communications software for transmitting data and for recording transaction logs. The library includes software for monitoring network processes, automatically warning system operators of potential problems, restarting processes that have failed, or migrating network services to an available server. For instance, the laboratory has configured multiple web servers with Linux Virtual Server (LVS) software for high-availability web, application and database service provisioning as well as load balancing. A round-robin balancing algorithm is currently used such that if the processing load on one server is heavy, incoming requests, be it HTTP, JSP or MySQL, are forwarded to the next available server by the LVS software layer. Listeners on one virtual server monitor the status and responsiveness of the others. If a failure is detected, an available server is elected as master and it assumes control and request forwarding for the entire LVS environment.

### Virtualized resources

Due to the rate that new servers need to be provisioned for scientific research, INI deploys a sophisticated high availability virtualized environment. This environment allows INI systems administrators to deploy new compute resources (virtual machines or VM's) in a matter of minutes rather than hours or days. Furthermore, once deployed, these virtualized resources can float uninhibitedly between all the physical servers within the cluster. This is advantageous because the virtualization cluster can intelligently balance virtual machines amongst all the physical servers, which permits resource failover if a virtual machine becomes I/O starved or a physical server becomes unavailable. The net benefit for LONI is more software resources are being efficiently deployed on a smaller hardware footprint, which results in a savings in hardware purchases, rack space and heat expulsion.

The software powering LONI virtualized environment is VMware's ESX 5. The ESX 5 is deployed on eight Cisco UCS B200 M3 servers, each with sixteen 2.6/3.3 GHz CPU cores and 128 Gb of DDR3 RAM. These eight servers reside within a Cisco UCS 5108 blade chassis with dual 8 × 10 Gb mezzanine cards providing a total of 160 Gb of available external bandwidth. Storage for the virtualization cluster is housed on the 23 nodes of Isilon storage. The primary bottleneck for the majority of virtualization solutions is disk I/O and the Isilon cluster more than meets the demands of creating a highly available virtualized infrastructure whose capabilities and efficiency meet or greatly exceed those of a physical infrastructure. A single six rack unit (6RU), eight blade chassis can easily replicate the resources of a 600+ server physical infrastructure when paired with the appropriate storage solution such as the INI Isilon storage cluster.

### Workflow processing

**Figure 2:**
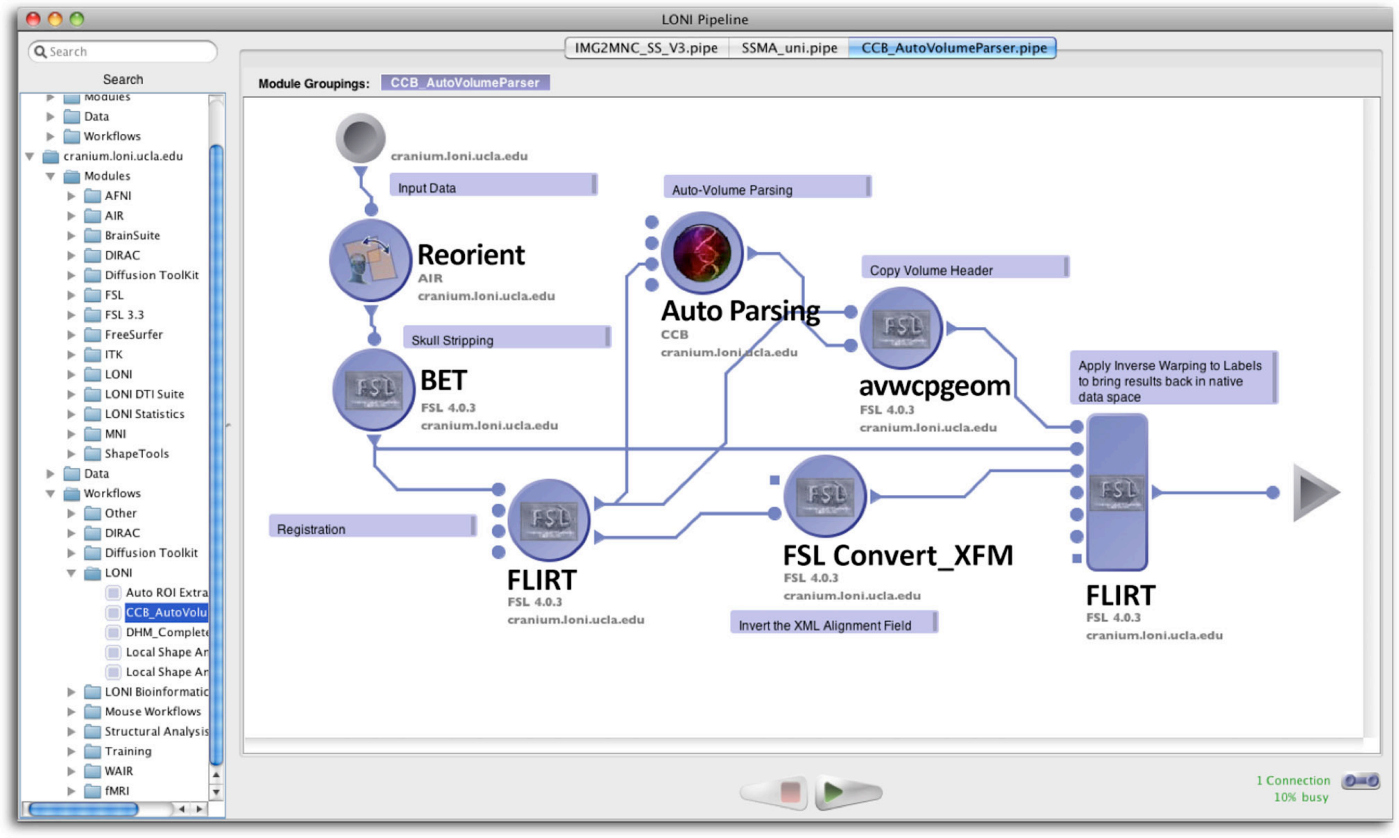
**The client interface to the LONI Pipeline execution environment**.

To facilitate the submission and execution of compute jobs in this compute environment, various batch-queuing systems such as SGE (https://arc.liv.ac.uk/trac/SGE) can be used to virtualize the resources above into a compute service. A grid layer sits atop the compute resources and submits jobs to available resources according to user-defined criteria such as CPU type, processor count, memory requirements, etc. The laboratory has successfully integrated the latest version of the LONI Pipeline (http://pipeline.loni.usc.edu) with SGE using DRMAA and JGDI interface bindings (Dinov et al., 2009, 2010; Torri et al., 2012). The bindings allow jobs to be submitted natively from the LONI Pipeline to the grid without the need for external scripts. Furthermore, the LONI Pipeline can directly control the grid with those interfaces, significantly increasing the operating environment's versatility and efficacy, and improving overall end-user experience. Figure 2 illustrates the latest version of the pipeline client software.

The data center will be approximately 3000 square feet and is being designed using cutting edge high density cooling solutions and high density bladed compute solutions. A total of 48 racks will be installed and dedicated to research use. Of the 48, 10 racks will be reserved for core services. The core services are on separate, dedicated, redundant power to ensure continuous operation. The current design of the data center includes a Powerware 9395 UPS system providing two 750 kW/825 kVA UPSs in a N+1 configuration for non-core racks and two 225 kW/250 kVA in a 2N configuration for core services racks. The UPS sends conditioned power to 300 kVA Power Distribution Units (PDUs) located inside the data center. The PDUs feed 400 A rated Track Power Busways mounted above rows of racks providing an “A” bus and a “B” bus for flexible overhead power distribution to the racks. The design calls for the use of VRLA batteries with 9 min of battery run time for the core services UPS and 6 min of battery run time for the non-core UPS (note that the generator requires less than 2 min of battery run time in order to fully take over the load in the event of an outage). A 750 kW/938 kVA diesel emergency generator located in a weatherproof sound attenuated enclosure adjacent to the building will provide at least 8 h of operation before needing to be refueled.

The Cisco UCS blade solution described above allows LONI to run the services of a much larger physical infrastructure in a much smaller footprint without sacrificing availability or flexibility. Each Cisco chassis hosts 8 server blades and has 160 Gb of external bandwidth available per chassis. Each of the 48 racks can hold up to 6 chassis plus requisite networking equipment (4 fabric extenders). Thus, the new data center has adequate rack space to accommodate this project.

In addition to a new data center, the INI infrastructure will house a 50-seat high definition theater—the Data Immersive Visualization Environment (DIVE). The prominent feature of the DIVE is a large curved display that can present highly detailed images, video, interactive graphics, and rich media generated by specialized research data. The DIVE display will feature a dominant image area, with consistent brightness across the entire display surface, high contrast, and 150° horizontal viewing angle. The display resolution target is 4 k Ultra HD, 3840 × 2160 (8.3 megapixels), in a 16:9 aspect ratio. Due to the ceiling height requirements, the DIVE will require two floors of the building. The DIVE is designed to facilitate research communication, dissemination, training, and high levels of interaction.

## Exemplary studies

### Alzheimer's disease imaging-genetics study

**Figure 3:**
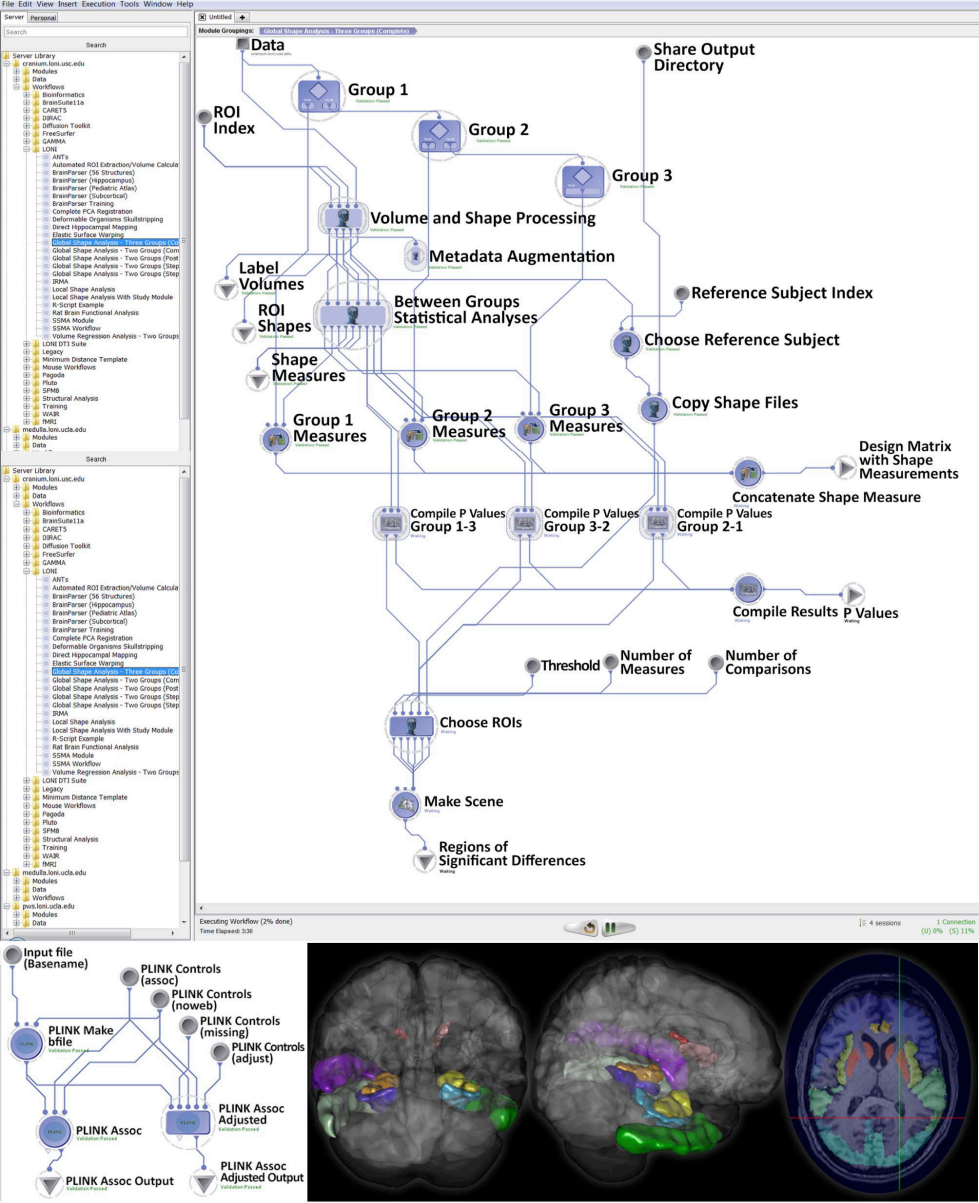
**Global shape analysis (GSA) protocol extracting neuroimaging biomarkers for each of the 3 cohorts (top), genetic phenotyping (bottom left), and examples of intermediate derived neuroimaging biometrics (bottom right)**.

Using subjects over the age of 65 from the Alzheimer's Disease Neuroimaging Initiative (ADNI) archive, http://adni.loni.usc.edu (Weiner et al., 2012), we investigated cognitive impairment using neuroimaging and genetic biomarkers. Querying the ADNI database, we selected 808 participants including 200 Alzheimer's Disease (AD) patients (108 males and 92 females), 383 mild cognitive impairment (MCI) subjects (246 males and 137 females), and 225 asymptomatic normal control (NC) volunteers (116 males and 109 females). After downloading the individual ADNI imaging data we carried standard quality control genetic analysis, using PLINK version 1.09, (Purcell et al., 2007). All data analytics were performed using the LONI Pipeline environment (Dinov et al., 2010; Torri et al., 2012). The global shape analysis protocol provides a set of 20 derived neuroimaging markers (*P* < 0.0001, between group ANOVA), which are studied in the context of the 20 most significant single nucleotide polymorphisms (SNPs), chosen by Manhattan plot, associated with the AD, MCI, and NC cohorts, as subject phenotypes. The structural ADNI data (1.5T MRI) were parcellated using BrainParser (Tu et al., 2008). The complete data analysis protocol and some of the intermediate results are shown on Figure 3.

**Figure 4:**
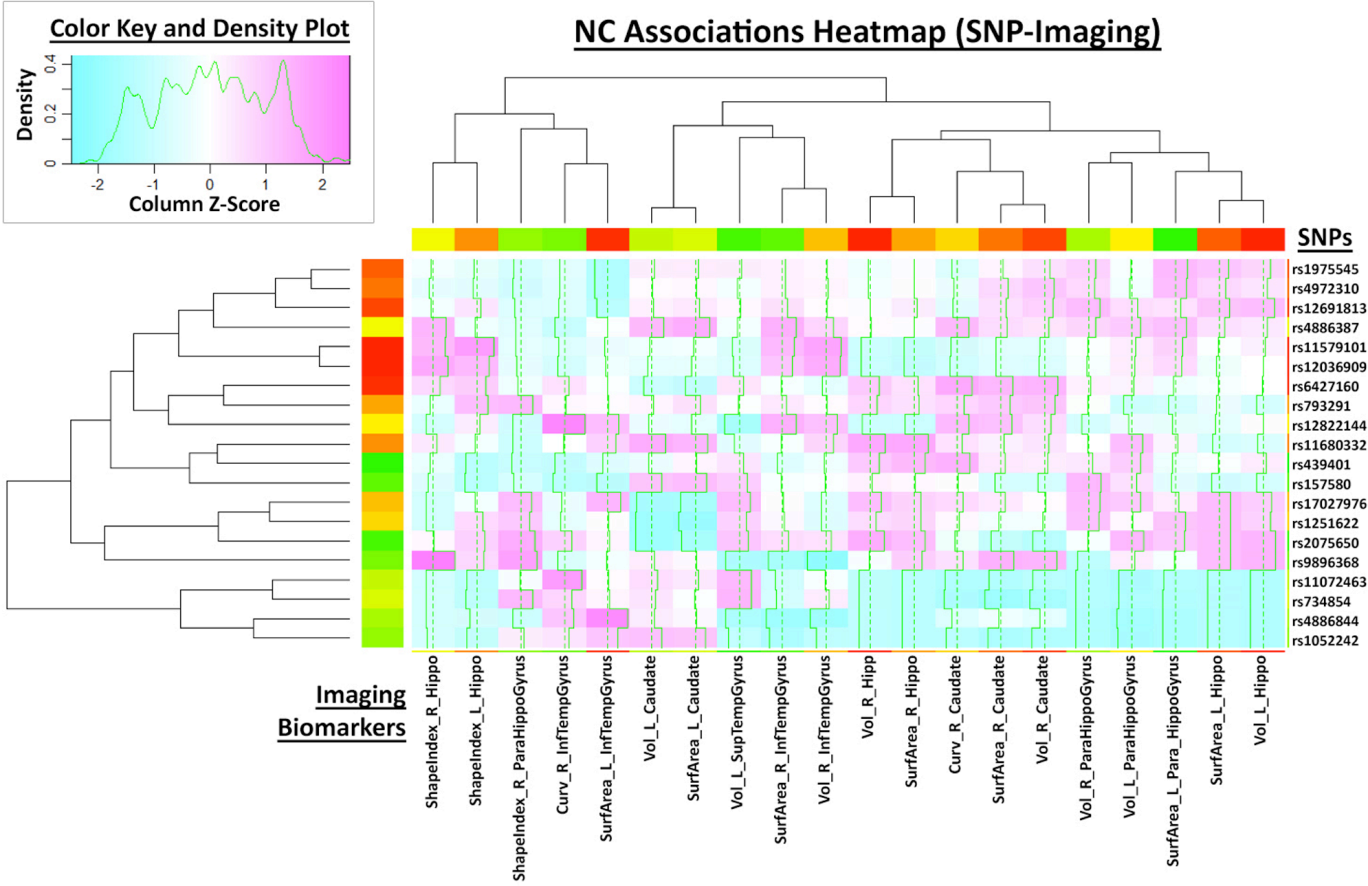
**Heatmap plot of 20 neuroimaging derivative measures associated with the subject phenotype (columns) and the SNP genotypes**.

This large scale study identified that neuroimaging phenotypes were significantly associated with the progression of dementia from NC to MCI and ultimately to AD. Our results pooling MCI and AD subjects together (*N*_1_ = 583) compared to NC subjects (*N*_2_ = 225) indicates significant association between 20 SNPs and 2 neuroimaging phenotypes as shown in the heatmap plot, Figure 4. The data analytics presented in this case study demand significant data storage, processing power and bandwidth capabilities to accomplish the end-to-end data processing, analysis and visualization. In this study the protocol includes about 100 independent processing steps and the analysis tool 2 days on a 1200 compute node shared cluster.

### Parkinson disease analytics

**Figure 5:**
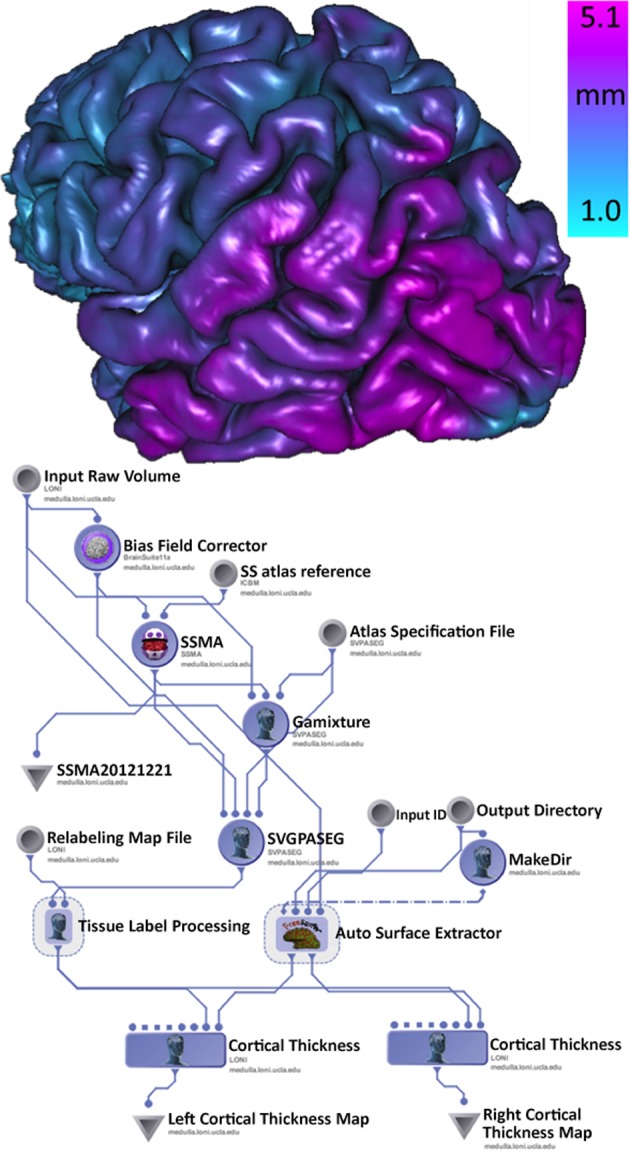
**Pipeline workflow protocol for automated extraction of imaging biomarkers and association of imaging and phenotypic PPMI data (left), and a 3D rendering of the cortical surface, colored by the gray matter thickness map, for one individual (right)**.

There is some clinical evidence that the different subtypes of Parkinson's disease (PD) may follow different clinical courses. Tremor-dominant cohorts show a slower progress of the disease and less cognitive decline than akinetic rigid group (Kang et al., 2013). The clinical subtypes probably are in concordance with differences in brain biochemical abnormalities. In this example, using the Parkinson Progression Marker Initiative (PPMI) brain data (Marek et al., 2011), we analyze structural brain changes in Parkinson's disease relative to their relationship with subtypes of Parkinson's disease. Specifically, the goal was to utilize the INI/LONI computational infrastructure to study interrelations between subtypes and biomedical imaging features in 150 PPMI subjects. This analysis protocol includes automatic generation of 56 regions of interest (ROIs) for each subject and computing various volume-based and shape-based measures for each region of interest (ROI), e.g., volume, dice coefficient, overlap measure, mean curvature, surface area, mean fractal dimension, shape-index, curvedness) (Dinov et al., 2010). Figure 5 illustrates a high-level view of the morphometric analysis of the data (left) and an example of an automatically generated gray matter thickness map for one of the processed cases. This workflow completed on the INI cluster in 2 days, competing with thousands of other processes that are run in parallel and submitted by different users.

Although both of these examples demonstrate a small fraction of the available processing modules and end-to-end computational workflow solutions, the Pipeline environment includes a much larger library of resources for image processing (Dinov et al., 2009), shape analysis (Dinov et al., 2010), next generation sequence analysis (Torri et al., 2012), and bioinformatics. These examples were chosen as they indicate demand for significant computational power to process hundreds of cases in parallel, the ability to handle high-throughput data transfer (near real time) with access to external databases, and the software necessary to pre-process, model, integrate, and visualize large multivariate datasets.

## Discussion

The neuroscience of the Twentieth Century was built upon the Popperian ideal of forming questions suitable as empirical hypotheses to be tested using experimentally derived data. Yet, with modern neuroimaging and genomics technologies, we are now able to gather more data per experiment that was gathered in perhaps years of collection 20 years ago. While the philosophy of science ideal based on hypothesis testing has by no means been surpassed, it is clear that the data being obtained offers greater information beyond the hypotheses under test which, indeed, offers more opportunity to explore larger data spaces and therefore form new testable lines for scientific investigation. Thus, in as much as the question itself is the driver of scientific progress, the data being obtained provides the chance to identify new questions worthy of our attention. For which, we will need to gather still more data.

As large quantities of data are gathered for any particular experiment, their accumulation into local databases and publicly available archives (e.g., the LONI Image and Data Archive; http://ida.loni.usc.edu) there is an increasing need for large-scale computational resources such as those discussed above. Be these resources local or remote (“in the cloud”), their availability helps to expedite data analysis, synthesis, its mining, and summarization such that old questions can be readily addressed and new questions can be formulated. With the increases in data size come increased needs to process data faster. The LONI/INI computational systems are one such example of pushing processing capability to the forefront of neuroimaging and genomic analytics. Other resources include Amazon, Microsoft, and Google services which, for a fee, users can provision data storage and multi-processor virtual systems upon which to configure and perform neuroimaging or genetics analyses. Irrespective of the form the computational infrastructure takes, there is little question that such services are a necessary element for Twenty-first Century biomedical science where data is king.

Data management including archival, query, retrieval, aggregation, and fusion are enabled via the LONI Pipeline Environment. For example, the initial data-sources within each workflow can pull data from different servers, aggregate it into the computational workflow, jointly process it and save intermediate and final results in different locations. As there is a growing array of publicly available data sets, this functionality is critical for large-scale collaborative studies requiring significant sample-sizes to identify associations and relations for (marginal) effect-sizes. Pipeline cloud-based data sources (inputs) and sinks (outputs) are similar to regular data sources and sinks, except that data are stored in the cloud. The Pipeline takes care of the data transfer between the cloud vendor and the compute nodes. Currently supported cloud source vendors include Amazon S3 and Dropbox (http://pipeline.loni.usc.edu/learn/user-guide/building-a-workflow/#Cloud%20sources%20and%20sinks). In addition, users can set up instances replicating the entire Pipeline infrastructure on Amazon EC2 (http://pipeline.loni.usc.edu/products-services/pipeline-server-on-ec2/).

The INI/LONI infrastructure has been specifically designed to meet the big data storage and processing challenges as evident from large-scale, multi-site neuroimaging initiatives such as ADNI, the Autism Centers of Excellence (ACE), PPMI, the Human Connectome Project (Toga et al., 2012), and others. With new NIH programs for brain research on the horizon, the computational systems and processing capabilities described here will find immediate application for the archiving, processing, and mining of vast quantities of neuroscience data from healthy as well as diseased subjects. There are several alternative Cloud-based computational neuroscience resources with similar goals and infrastructure. For example, the Neuroscience Gateway (www.nsgportal.org) portal is supported by the Extreme Science and Engineering Discovery Environment (XSEDE) Resource Allocation Committee and provides High Performance Computing resources for the neuroscience community. The Neuroimaging Tools and Resources Clearinghouse (NITRC) Amazon EC2 Computational Environment is a virtual computing platform configured with many neuroimaging data analysis applications (https://aws.amazon.com/marketplace/pp/B00AW0MBLO?sr=0-2). The INI/LONI infrastructure does have its limitations. System bottlenecks include potential for large number of simultaneous users (hundreds), or a few heavy users (e.g., a dozen users with complex protocols involving tens of thousands of jobs managed in parallel), can significantly impact the performance of the back-end Pipeline server and NFS manager. Data I/O access could be affected when managing a huge number of simultaneous read-write requests, including handling intermediate results. Upgrading the infrastructure (e.g., hardware expansions, system updates, software upgrades) require a significant concerted effort.

INI/LONI welcomes new ideas from the entire computational community and constantly promotes new collaborations with outside investigators. The LONI/INI infrastructure is freely available to the entire community (registration and accounts are required). There is a variety of data, modeling, computational, scientific or translational-research collaborations we support, which can be initiated by completing one of the online web-forms (http://resource.loni.usc.edu/collaboration/collaborator-application/). This manuscript attempts to demonstrate how the entire biomedical community can utilize the LONI resources, as well as demonstrate the design-challenges, capabilities, and maintenance of such integrated data, software and hardware architectures, which may be valuable to others interested in building similar, alternative or federated computational frameworks.

### Conflict of interest statement

The authors declare that the research was conducted in the absence of any commercial or financial relationships that could be construed as a potential conflict of interest.
